# NAFLD-Related HCC: Focus on the Latest Relevant Preclinical Models

**DOI:** 10.3390/cancers15143723

**Published:** 2023-07-22

**Authors:** Jing Fang, Séverine Celton-Morizur, Chantal Desdouets

**Affiliations:** 1 Centre de Recherche des Cordeliers, INSERM, Sorbonne Université, Université de Paris, 75006 Paris, France; jing.fang@inserm.fr; 2 Genomic Instability, Metabolism, Immunity and Liver Tumorigenesis Laboratory, Equipe Labellisée Ligue Contre le Cancer, 75005 Paris, France

**Keywords:** non-alcoholic fatty liver disease, non-alcoholic steatohepatitis, hepatocarcinoma, lipid, inflammation, fibrosis, preclinical mouse models, and in vitro human cell models

## Abstract

**Simple Summary:**

Non-alcoholic fatty liver disease (NAFLD) is a spectrum of disease ranging from simple steatosis (NAFL) to non-alcoholic steatohepatitis (NASH), which can progress to fibrosis and cirrhosis and ultimately lead to hepatocarcinoma (HCC). Due to its increasing prevalence, NAFLD has become a major public health problem. Given the partial understanding of the complex pathological mechanisms of NAFLD-induced human HCC and the lack of effective treatment, relevant pre-clinical models are still urgently needed to better recapitulate and investigate the process and mechanism of human NAFLD/HCC. This review discusses a selection of the most relevant mouse models in the study of NAFLD/HCC, with their specific advantages and disadvantages, and also the emergence of new ex vivo technologies, which will greatly accelerate the transition from basic science to clinical discoveries.

**Abstract:**

Hepatocellular carcinoma (HCC) is the most common type of primary liver cancer and one of the deadliest cancers worldwide. Despite extensive research, the biological mechanisms underlying HCC’s development and progression remain only partially understood. Chronic overeating and/or sedentary-lifestyle-associated obesity, which promote Non-Alcoholic Fatty Liver Disease (NAFLD), have recently emerged as worrying risk factors for HCC. NAFLD is characterized by excessive hepatocellular lipid accumulation (steatosis) and affects one quarter of the world’s population. Steatosis progresses in the more severe inflammatory form, Non-Alcoholic Steatohepatitis (NASH), potentially leading to HCC. The incidence of NASH is expected to increase by up to 56% over the next 10 years. Better diagnoses and the establishment of effective treatments for NAFLD and HCC will require improvements in our understanding of the fundamental mechanisms of the disease’s development. This review describes the pathogenesis of NAFLD and the mechanisms underlying the transition from NAFL/NASH to HCC. We also discuss a selection of appropriate preclinical models of NAFLD for research, from cellular models such as liver-on-a-chip models to in vivo models, focusing particularly on mouse models of dietary NAFLD-HCC.

## 1. Introduction

Non-alcoholic fatty liver disease (NAFLD) is becoming the most common cause of chronic liver disease worldwide and is thus a public health problem of great concern [1,2,3,4,5,6]. NAFLD is associated with several metabolic disorders, such as obesity, type 2 diabetes mellitus, hypertension, and dyslipidemia. The prevalence of NAFLD is currently estimated at 25–35% in the general population, up to 50% in type 2 diabetes patients, and up to 90% in morbidly obese patients [6,7,8]. This disease encompasses a spectrum of liver conditions, ranging from simple hepatic steatosis or non-alcoholic fatty liver (NAFL) to the concomitant presence of hepatocellular damage (ballooning), Mallory-Denk body formation, and lobular necro-inflammation, defining non-alcoholic steatohepatitis (NASH), which can lead to various degrees of fibrosis [9,10,11,12]. NAFL is associated with a low risk of adverse outcomes, but the chronic liver injury occurring in NASH markedly increases the risk of end-stage liver diseases, such as cirrhosis and hepatocellular carcinoma [1,13,14]. The incidence of NAFLD-related HCC is highly variable [15]. The estimated annual incidence of HCC ranges from 0.5% to 2.6% among patients with NASH cirrhosis. HCC can develop in NAFLD patients without cirrhosis [1], and it has been estimated that almost half of the patients with NAFLD-related HCC have no marked liver fibrosis [16,17,18]. This clinical observation poses a challenge for HCC surveillance. The epidemiological features of HCC are changing, with the increase in vaccination coverage for HBV and the effective antiviral therapy for HCV infection decreasing the burden of virus-associated liver cancer worldwide [13]. Given the global increase in obesity and type 2 diabetes, it has been predicted that NAFLD will become the most common underlying etiological risk factor for HCC [13,19]. Unsurprisingly, NASH is already one of the major indications for liver transplantation worldwide [20,21,22]. There is currently no effective treatment for NAFLD, and most management approaches are based on preventive measures involving regular physical activity, low-calorie eating, and weight loss [23]. NAFLD is a complex disease, the development and progression of which require multi-hit combinations of various risk factors, including age, sex, ethnicity, dietary habits, hormonal status, genetic predisposition (e.g., polymorphisms of *PNPLA3*, *TM6SF2*, *MBOAT1*, or *HSD17B13*), epigenetic factors, and associated comorbid conditions (e.g., obesity, type 2 diabetes mellitus, obstructive sleep apnea, and gut dysbiosis) [4,5,24,25,26]. The individual impacts of these factors probably change over time, determining the phenotype and natural course of the disease.

Triglyceride accumulation (e.g., steatosis) is believed to be relatively benign, whereas hepatocyte lipotoxicity results chiefly from the accumulation of free fatty acids, free cholesterol, and other lipid metabolites [5,27,28]. These changes within the liver place extra metabolic stress on various organelles, such as the mitochondria and endoplasmic reticulum, triggering a cascade of stress-induced responses, including the generation of reactive oxygen species (ROS) [29,30,31]. This leads to further cell injury and programmed cell death via apoptosis, necrosis, or necroptosis, with the release of damage-associated molecular patterns (DAMPs) [32]. Cellular senescence has also been identified as a major actor in NAFLD progression through changes in mitochondrial β-oxidation and the release of inflammatory signals, the so-called “senescence-associated secretory phenotype”, in particular [33,34]. Local inflammation mediated by Kupffer cells and the influx of platelets, neutrophils, and inflammatory monocytes have been shown to play major roles in the inflammatory mechanism [35,36,37,38]. Over time, various adaptive immune cells (e.g., CD8^+^PD^−^1^+^ T cells) and innate immune cells (e.g., CXCR1^+^ cDCs) infiltrate the liver, supporting the development of autoaggressive CD8^+^ T cells [35,39].

The development of experimental models accurately reproducing the mechanisms underlying human NAFLD remains highly challenging. Nevertheless, diverse in vitro and in vivo models of NAFLD have been developed and have significantly advanced our understanding of NAFLD’s pathophysiology, making it possible to test new therapeutic targets [40,41,42,43]. In this review, we focus on the most widely used preclinical mouse models of NAFLD and in vitro human cell models of NAFLD crucial for drug development.

## 2. Mouse Models of Non-Alcoholic Fatty Liver Disease

**Figure 1:**
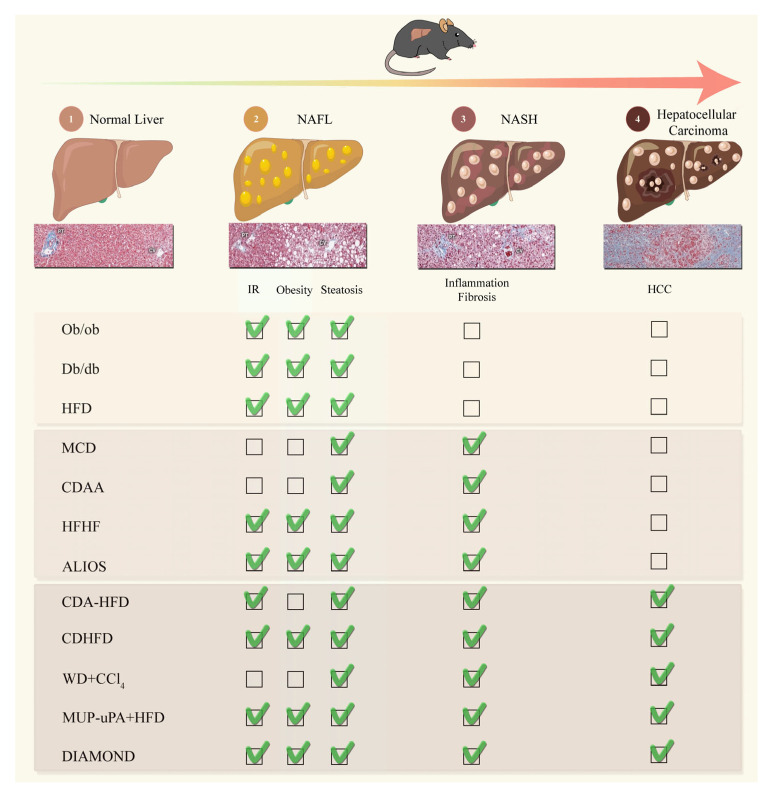
Pre-clinical models mimicking the physiopathological characteristics of Non-Alcoholic Fatty Liver Disease/HCC. ALIOS: American-lifestyle-induced obesity syndrome; CCl_4_: Carbon tetrachloride; CDAA: Choline-deficient, L-amino defined diet; CDA-HFD: Choline-deficient, L-amino acid-defined, high-fat diet; CD-HFD: Choline-deficient, high-fat diet; Db/db: leptin-receptor-deficient; DIAMOND: Diet-induced animal model of non-alcoholic fatty liver disease; HCC: Hepatocellular carcinoma; HFD: High-fat diet; HFHF: High-fat high-fructose diet; MCD: Methionine and choline deficient diet; MUP-uPA: major urinary protein-urokinase-type plasminogen activator; NAFL: Non-alcoholic fatty liver; NASH: Non-alcoholic steatohepatitis; Ob/ob: leptin deficient; and WD: Western diet.

NAFLD is considered to be a multiple-hit multisystem disease. This complexity makes it difficult to develop comprehensive models that fully reproduce the mechanisms of human NAFLD. Over the last two decades, investigators have addressed key questions about NAFLD with various rodent models, mostly in mice (Figure 1).

### 2.1. Murine Models of NAFL

#### 2.1.1. High-Fat Diet (HFD)

One of the most widely used methods for obtaining overweight animals and inducing steatosis is the administration of a high-fat diet (HFD), generally with fats accounting for 45 to 60% of the total calorie content [44,45]. The fats included in this diet may include saturated, monosaturated, or polysaturated fatty acids, separately or in various combinations. HFD-induced metabolic syndrome in rodents can be modified by many factors, including the duration of the dietary exposure and the species, strain, age, and sex of the animals [46]. Mice fed a HFD for at least 10 to 14 weeks generally develop hyperlipidemia, insulin resistance, and glucose intolerance, the key features of metabolic syndrome and obesity in humans [44,47,48]. Their hepatocytes display fat accumulation, ballooning, and Mallory-Denk bodies. After nine months on a HFD, mice may display a significant increase in their circulating levels of liver enzymes, such as alanine aminotransferase (ALT) and aspartate aminotransferase (AST) [44,49]. However, they present only minor signs of inflammation and fibrosis [49,50]. Velazquez and coworkers investigated the effect of prolonged feeding on a HFD (80 weeks). They reported a significant increase in steatosis, inflammation, cell injury, fibrosis, and ER stress, mimicking the effects on the microbiota observed in NAFLD patients [51]. Mice fed a HFD are generally considered to be a model of non-alcoholic fatty liver (NAFL).

#### 2.1.2. Lepob/Lepob (*ob/ob*) Mice

Leptin, a peptide hormone secreted principally by white adipose tissue, acts via the hypothalamus to decrease food intake and increase energy expenditure [52,53]. *Lepob/Lepob* (*ob/ob*) mice carry an autosomal recessive mutation of the leptin gene. Homozygosity for this mutation results in a redistribution of fat from the adipose tissue to the liver and other non-adipose tissues. *Ob/ob* mice are hyperphagic, inactive, sluggish, and gradually become severely obese, hyperlipidemic, hyperglycemic, hyperinsulinemic, and insulin-resistant with age (3–4 weeks old) [53]. In *ob/ob* mice, lipotoxicity and lipoapoptosis are induced in the hepatocytes within the liver parenchyma. However, these obese mice rarely develop severe liver damage or fibrosis, and do not, therefore, develop spontaneous steatohepatitis [54,55]. Nevertheless, NASH without progressive fibrosis can be induced by a second hit, such as a non-chow diet (e.g., MCD diet) or exposure to small doses of lipopolysaccharide endotoxin or ethanol [56,57,58].

#### 2.1.3. Leptin-Receptor-Deficient (*db/db*) Mice

*Db/db* mice, or leptin-receptor-deficient mice, are homozygous for an autosomal recessive point mutation of a diabetes gene (*db*), resulting in an absence of the long isoform of the leptin receptor [49,59,60]. Leptin levels are normal or high in *db/db* mice, but their leptin signaling is defective [49,61]. The phenotype of *db/db* mice, which have an abnormally strong appetite, obesity, hyperglycemia, hyperinsulinemia, insulin resistance, and macrovesicular hepatic steatosis, is very similar to that of *ob/ob* mice. However, microvesicular steatosis, a feature of impaired mitochondrial function [62], is more frequently observed in *db⁄db* mice than in *ob/ob* mice [61]. The development of NASH features also requires additional stimulation in *Db/db* mice [61,63]. Interestingly, after three months on a high-calorie diet, *db/db* mice present steatohepatitis (NAS ≥ 5), whereas *ob/ob* mice do not develop NASH in the same conditions [61]. Iron overload in *db/db* mice has been shown to induce key features of NASH, such as an increase in hepatic oxidative stress and fibrogenesis [64].

### 2.2. Murine Models of NASH

#### 2.2.1. Methionine- and Choline-Deficient Diet (MCD)

The MCD model is one of the most widely used dietary models in NASH research. The MCD contains a high proportion of sucrose (40%), moderate amounts of fat (10%), and is deficient in methionine and choline [65,66]. Choline is the precursor of phosphatidylcholine, which is essential for very-low-density lipoprotein (VLDL) production. Methionine plays a major role in the synthesis of glutathione, a crucial antioxidant protein. The MCD induces NAFLD through high levels of fatty acid intake and low levels of VLDL secretion in the context of strong oxidative stress. After four to six weeks on the MCD, the liver parenchyma presents steatosis, hepatocyte ballooning, cell death, inflammation, oxidative/ER stress, and fibrosis [43,66], without the development of insulin resistance or related comorbid conditions [67,68]. One of the drawbacks of using the MCD to induce NASH development is the severe weight loss and liver atrophy caused by this diet, which are not characteristics of human NASH [69,70]. As with the HFD, the sensitivity to the MCD differs between mouse strains, with A/J mice displaying significantly higher serum alanine aminotransferase (ALT) levels and a greater weight loss than other strains [71]. The MCD model mimics the histological phenotype of human NASH relatively quickly, but the use of this model is limited by the known metabolic profile disparities of this model relative to human NASH [43].

#### 2.2.2. Choline-Deficient L-Amino Acid-Defined (CDAA) Diet

The CDAA diet contains 30% fat in terms of calorie content (mostly Primex fat as a source of *trans* fat) and 0.17% methionine to compensate for the choline deficiency [72,73]. Histological analyses of liver samples from C57BL/6J mice fed with the CDAA diet revealed steatosis (score 3) and focal lobular inflammation (score 1) after six weeks on the diet, followed by mild features of NASH after 12 weeks on the diet. Chronic feeding on this diet for at least 20 weeks is required for the development of fibrosis [74]. As in the MCD model, mice on the CDAA diet do not gain weight and do not develop insulin resistance [75,76]. After long-term feeding on this diet (65–84 weeks), male mice develop hepatocellular preneoplastic foci, adenomas (incidence: 65%), and carcinomas (incidence: 25%) associated with fibrosis and oxidative DNA damage [75]. It remains unclear whether the progression of NASH with fibrosis alone is sufficient to influence HCC development, or whether aging is also involved. These findings suggest that this model, like the MCD model, displays marked differences from human NAFLD.

#### 2.2.3. High-Fructose Diet

There is now clear evidence that high-fructose diets are a major risk for the development of obesity and NAFLD [77,78]. A number of studies on rodent models have evaluated the influence of fructose on NAFLD development through the addition of fructose to their drinking water (55% fructose) or directly to their pelleted diet (60% fructose) [79,80,81]. C57BL/6 mice fed with a high-fat diet, without (HFD) or with high (HFHF) fructose in their drinking water, presented similar increases in body weight, body fat mass, and fasting glucose after 16 weeks, and both groups of mice displayed insulin resistance relative to mice fed with standard chow [80]. However, the HFHF mice displayed an increase in hepatic ROS production and a NASH-like phenotype, with significant fibrosis not observed in the HFD mice. The underlying mechanisms for this fibrosis seemed to involve the induction, by fructose, of an increase in ROS production, associated with CD11b^+^F4/80^+^Gr1^+^ hepatic macrophage aggregation, resulting in transforming growth factor beta-1 signaling-mediated collagen deposition [80]. Nigro and coworkers showed that fructose consumption also induces an amplifying loop involving lipogenesis, palmitate, Nrf2, and Nlrp3, leading to a higher risk of NAFLD progressing to NASH [81]. Fructose has been shown to induce alterations to the tight junction proteins that affect the gut permeability, with the translocation of bacteria and bacterial endotoxins into the bloodstream [82]. Karin and coworkers recently showed that a short-term high-fructose diet had no effect on intestinal permeability. However, long-term feeding on such a diet induced barrier deterioration and intestinal epithelial ER stress [83]. Notably, fructose-elicited endotoxemia activated Toll-like-receptor (TLR) signaling and TNF production via liver macrophages, inducing lipogenic enzymes [83].

#### 2.2.4. The American-Lifestyle-Induced Obesity Syndrome (ALIOS) Diet

The American-lifestyle-induced obesity syndrome (ALIOS) mouse model is a dietary intervention based on the nutritional content of fast foods commonly consumed in the Western world [84]. Mice are fed with high-fat chow (45%), including *trans* fats, with high-fructose corn syrup added to their drinking water. The animals become obese, have a high HOMA index and impaired glucose tolerance, and develop hepatic steatosis, with a necroinflammatory response and fibrosis occurring within 16–26 weeks [84,85]. This diet has been demonstrated to reproduce the histopathological characteristics of human NASH [84,85]. Interestingly, the ALIOS diet induces NASH in both male and female rodents [86]. Hepatic transcriptomic analyses have revealed changes in the expression of multiple genes associated with inflammation and tissue repair in ALIOS-diet-fed mice [86]. In the past, this diet was widely used to model liver dysfunction, but its availability has decreased due to the current ban on *trans* fats. Alternatives to the ALIOS diet are currently being explored, including the Gubra Amylin NASH model and Non-Trans Fat Western Diet (WD-NTF), which has yielded promising results for the induction of NASH in mouse models (for a review see [43]).

### 2.3. Murine NASH-HCC Models

#### 2.3.1. Choline-Deficient L-Amino Acid-Defined, High-Fat Diet (CDA-HFD)

The CDA-HFD (choline-deficient, L-amino acid-defined, high-fat diet, with 60% of the calorie content as fat and 0.1% methionine by weight) model resembles human NAFLD more closely than the CDAA diet model [76]. The CDA-HFD induces IR, increases hepatic steatosis, and alters the levels of the enzymes involved in carbohydrate and lipid metabolism. Histological changes similar to those seen in human NASH are observed, including hepatocyte ballooning and severe fibrosis [76,87]. After 24 weeks on this diet, an increase in the expression of carcinoembryonic markers (e.g., Afp and Gpc3) is observed, and HCC develops after 36 weeks [87,88]. Even after switching back to a standard diet at 37 weeks, many mice display a progression of fibrosis, with the development of HCC at 48 weeks [88]. Interestingly, NAFLD development clearly differs between the sexes in this diet-induced model. CDAHFD-fed male and female mice initially display similar hepatic damage after 6 weeks on the diet, but differences between the sexes are observed after 12–36 weeks. Male mice fed the CDAHFD present more severe hepatic damage, with greater TG accumulation, hepatocyte death, inflammation, fibrosis, and even tumorigenesis, in comparison to female mice fed the same diet [89]. This higher prevalence of NASH/HCC in male mice matches observations in humans [90]. Lee and collaborators recently demonstrated that formyl peptide receptor 2 (FRP2), an important mediator of inflammatory and immune responses [91], mediates the sex-specific responses to CDAHFD-diet-induced NAFLD/NASH [89].

#### 2.3.2. Choline-Deficient, High-Fat Diet (CD-HFD)

Mathias Heikenwalder and coworkers used a choline-deficient high-fat diet (CD-HFD) to study the combined long-term effects of this diet on obesity/metabolic syndrome/NASH/HCC [35,39,43]. Male C57BL6/J mice fed with the CD-HFD presented progressive body weight gain, glucose intolerance, and insulin resistance. After 16 weeks on the CD-HFD, the mice displayed ballooning hepatocytes, ER/oxidative stress, immune cell infiltration, satellitosis, Mallory-Denk body (MDB) formation, and glycogenated nuclei, all of which are features of human NASH. This diet induced the activation of intrahepatic CD8(+) T cells, NKT cells, and inflammatory cytokines, as observed in NASH patients [35]. Only mild fibrosis developed in this model. The females gained less weight than the males and presented milder liver damage, steatosis, and inflammation [35]. Prolonged exposure to the CD-HFD leads to tumor development. Macroscopically visible tumors were detected in ~25% of mice after 12 months on the diet, and in ~50–70% of mice after 15 months [35]. Using this model, Malehmir et al. demonstrated that platelet recruitment to the liver contributes to the development of nonalcoholic steatohepatitis (NASH) and hepatocellular carcinoma (HCC) via platelet glycoprotein Ibα (GPIbα) [92]. Moreover, Pfister et al. showed that CD8^+^PD1^+^ T cells have a pro-tumorigenic effect in NASH, driving HCC formation [39]. This model is also particularly suitable for testing the efficacy of HCC treatments [39,92].

#### 2.3.3. Western Diet (WD) + Carbon Tetrachloride (CCl_4_)

Carbon tetrachloride (CCl_4_) is a hepatotoxic chemical used to induce liver damage, fibrosis, and cirrhosis in experimental animals. Friedman and coworkers developed a mouse model of NASH with extensive fibrosis by combining a high-fat, high-fructose, high-cholesterol Western diet (WD) with weekly low-dose intraperitoneal CCl_4_ [93]. Histological analyses revealed that CCl_4_ exacerbated the hepatocyte ballooning, proliferation, inflammation, and fibrosis induced by the WD. In this model, NASH was induced within 12 weeks, with severe steatohepatitis, stage-three bridging fibrosis, and subsequent stage-four cirrhosis and HCC being observed by 24 weeks [93]. The dysregulation of the molecular pathways in WD/CCl_4_ mice at 12 weeks resembled that in early/mild human NASH [93]. Moreover, the HCCs collected from the WD/CCl_4_ mice at 24 weeks were similar to human HCC molecular subclasses, highlighting the ability of this model to parallel human disease progression [93]. This model has also been shown to be valuable as a system for investigating the NASH microbiome. Carter et al. revealed that microbiome remodeling was completed within 12 weeks in this model, consistent with the evidence of advanced fibrosis, hepatocellular injury, inflammation, and intestinal barrier dysfunction [94]. One limitation of this model is that the CCl_4_ treatment attenuated the increases in body weight, cholesterol, and insulin/glucose levels typically observed on the WD [93]. Nevertheless, these mice remain a good model, reproducing the progressive stages of human fatty liver disease, from simple steatosis to inflammation, fibrosis, and cancer.

#### 2.3.4. MUP-uPA + HFD

Karin and coworkers developed a model for NASH-driven HCC based on *MUP-uPA* transgenic mice fed with a HFD [40,50]. The *MUP-uPA* mice experienced transient hepatic ER stress early in life due to the high levels of uPA expression sustained by the HFD. The full range of NASH-like pathological features are induced in this model, including hepatocyte ballooning, inflammatory infiltrates, and pericellular and bridging fibrosis within four months on the diet, with continuous hepatocyte death and compensatory proliferation. High levels of TNF production, ER stress, and the hepatic expression of p62, all of which have been implicated in human disease, are involved in the progression of both NASH and HCC [50,95]. A spontaneous progression to HCC was observed in 60–85% of the male mice within 40 weeks of this diet. As these mice can be studied from the onset of NASH until they develop tumors, this model can be used for biomarker discovery and the identification of the molecular drivers of HCC progression. The immunopathogenesis of the progression of NASH to HCC in *MUP-uPA* mice is very similar to that in human NASH-driven HCC, with the precancerous liver accumulating PD-L1- and IL-10-expressing IgA^+^ cells in parallel with the appearance of inflammation-induced HCC progenitor cells [96]. HFD-fed MUP-uPA mice may be a model of choice for testing treatments. Feng He et al. recently showed that ferroptosis inhibitors or ATF4 activators may be useful for preventing the development of NASH and its progression to HCC [97].

#### 2.3.5. DIAMOND Mice

Sanyal and coworkers described a diet-induced animal model of NAFLD (DIAMOND) based on an isogenic strain derived from a cross between two common mouse strains, 129S1/SvImJ and C57BL/6J [98]. Mice (at 8–12 weeks of age) were fed with a high-fat, high-carbohydrate diet (WD, 42% of calories from fat and 0.1% cholesterol by weight) with ad libitum access to glucose/fructose in their drinking water. B6/129 mice developed obesity, insulin resistance, hypertriglyceridemia, and an increase in their LDL-cholesterol levels following the introduction of this diet. They subsequently also developed steatosis (4–8 weeks), steatohepatitis (16–24 weeks), progressive fibrosis (16 weeks onwards), and 90% went on to develop spontaneous HCC. The histopathological and transcriptional characteristics of DIAMOND mice resemble those of human NASH patients, with lipogenic, ER/oxidative stress, and the activation of the inflammatory and apoptotic signaling pathways [98]. Moreover, the transcriptomic HCC gene signature is similar to that of the S1 and S2 subclasses of human HCC [98,99].

## 3. Ex Vivo Models of Non-Alcoholic Fatty Liver Disease

**Figure 2:**
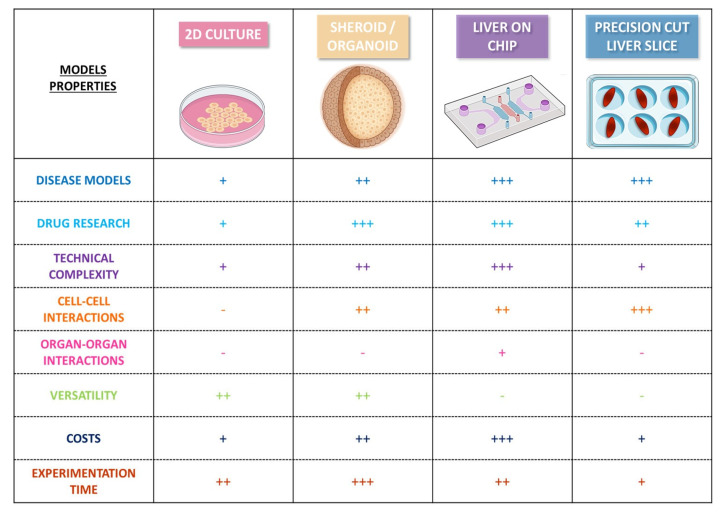
Human in vitro models of Non-Alcoholic Fatty Liver Disease. −: inadequate; +: low; ++: medium; and +++: high.

In recent years, the critical need to develop effective therapies for the treatment of NAFLD/NASH has led to the emergence of new in vitro models for studies of the mechanisms involved in the development of the disease and for drug screening. Until recently, basic models consisting of two-dimensional (2D) monolayer cultures of single cell types were used to mimic part of the pathogenic process in NAFLD (e.g., steatosis) and to investigate the lipid-lowering effects of anti-steatotic compounds [60,100,101]. However, it proved challenging to model NAFLD fully with such conventional 2D models, due to the chronic nature of the disease. The complete modeling of NAFLD requires long-term stable cultures and an intricate interplay between parenchymal and nonparenchymal liver cells to mediate the disease progression and inflammatory responses [102]. Efforts have been made to overcome these shortcomings by developing new models more closely resembling the architectural and functional properties of in vivo tissues and by promoting alternatives to animal experiments in line with the “3Rs” concept: three-dimensional (3D) models, such as spheroids, organoids, liver-on-chip, and precision-cut liver slices [60,101,103,104] (Figure 2).

### 3.1. Spheroids

Spheroids were first introduced in the early 1970s by Sutherland and coworkers [105] and are now among the most widely used 3D culture models. Spheroids are formed when simple clusters of cells stick to each other. Three-dimensional floating spheres can be obtained with or without the support of a scaffold [106]. Scaffold-free methods are widely used as they are relatively simple, inexpensive, and rapidly generate spheroids, with the single-cell suspension typically being maintained in ultra-low attachment plates [107]. Scaffold-based methods are generally used in tissue engineering and regenerative medicine applications [106]. Spheroids can remain viable in culture for long periods of time and can reproduce some of the functional properties of an organ [108,109]. Furthermore, depending on the aim of the research (e.g., drug safety screening or the development of antitumor strategies), specific protocols have emerged to obtain spheroids of specific dimensions, composed of cells in different proliferative and metabolic states [106,110,111].

Liver spheroids can be derived from immortalized hepatic cell lines (generally HepG2/HepaRG), differentiated embryonic stem cells (ESC), or pluripotent stem cells (PSC), but are mostly derived from primary human hepatocytes (PHH), which are generally obtained from human liver resections or non-transplantable organs [112]. PHH cultured as 3D spheroids are the model that most closely mimics the phenotype of a hepatocyte in vivo, as these spheroids maintain cell–cell interactions, a tissue-like architecture, and hepatocyte-specific functions over periods of culture of at least five weeks [108]. They have been successfully used to model and study the liver’s metabolic pathophysiology [108,113,114,115,116].

The simple supplementation of spheroids with a mixture of palmitic and oleic acids, two common dietary long-chain FFAs that accumulate in excess in the liver during human steatosis [117], reproduces hallmarks of NAFLD-NASH (e.g., cytoplasmic accumulation of TG in hepatocytes, ER stress, inflammation, and cell death [60,101,104,118]). Kozyra and coworkers described the use of spheroids cultured in a lipotoxic environment as a model for steatosis and insulin resistance [115]. They also demonstrated a reversal of hepatic steatosis following treatment with various antisteatotic agents (metformin, or the antioxidant vitamin E), providing support for the view that 3D spheroids open up new perspectives for studies of potential pharmaceutical targets [115]. Furthermore, the use of multilineage 3D spheroids (coculture of hepatocytes with non-parenchymal cells) was found to provide a better characterization of the relevant mechanistic steps connected to liver steatosis and fibrosis during the progression of NAFLD to NASH. Following exposure to FFA or cyclosporine A, cocultured spheroids displayed enhanced steatogenesis and collagen production, an upregulation of the genes associated with fibrosis progression, such as TIMP metallopeptidase inhibitor 1 (TIMP1) or actin alpha 2 (ACTA2), HSC activation, and the induction of pro-inflammatory cytokines [108,119,120]. Furthermore, intraspheroid steatosis and fibrosis were attenuated by treatment with drugs (Cenicriviroc, Liraglutide, Selonsertib, or Firsocostat) currently under evaluation in clinical trials for the treatment of NASH [119,120,121]. PHH spheroids have also been used in studies on the human genetic variants associated with altered lipid biosynthesis, the establishment and progression of steatosis [119,120,122,123], inflammation [124], and fibrosis [119,123], and have been proved promising for the investigation of clinically relevant associations in NAFLD.

The 3D liver spheroid system elicits many of the phenomena observed in vivo, such as insulin resistance and, importantly, the reversibility of steatosis, making this system suitable both for studies of NAFLD’s pathophysiology and for global therapeutic drug screening. However, size variability and the formation of larger aggregates, hindering both nutrient supply and oxygen diffusion [125], are major shortcomings that will need to be improved if spheroid models are to be more widely used.

### 3.2. Organoids

Until recently, the terms “organoid” and “spheroid” were used interchangeably [126], but these two entities are produced differently and originate from different cells. Organoids are three-dimensional assemblies of one or more cell types that partially resemble the organ modeled and can perform one or more of its functions [106,127]. Organoids can develop from stem cells (pluripotent, fetal, or adult) [127] or organ-specific progenitors [128,129] through a self-organization process [130].

Liver organoids are generated by the incorporation of tissue stem cells, progenitor cells, or tissue-resident cells isolated from liver samples into an extracellular scaffold environment, such as Matrigel or collagen [106,131]. Unlike induced pluripotent stem cell (iPSC)-derived liver organoids, which are generated by a stepwise differentiation process, human liver-tissue-derived organoids may proliferate and can be screened for gene function [132]. Human liver organoids consist of a spherical monolayer of polarized hepatocytes with a bile canaliculus-like architecture; they can maintain directional bile acid excretion for several weeks [133,134]. With a view of reproducing some of the key features of steatosis and steatohepatitis, Ouchi and coworkers used pluripotent stem cell lines to develop a multicellular human liver organoid (HLO) composed of hepatocyte-, stellate-, and Kupffer-like cells, with a transcriptome similar to that of the tissues from which they were derived in vivo [135]. The exposure of these triple-lineage iPSC-derived organoids to various doses of FFA resulted in a dose-dependent accumulation of intracellular lipids, increases in the secretion of inflammatory cytokines and collagen, and hepatocyte ballooning. The authors proposed organoid stiffness as a potential readout for evaluating the fibrosis severity and for a direct assessment of the efficacy of potential anti-fibrogenic drug candidates [135]. Personalized studies of liver function in patients with NAFLD/NASH-specific differences are essential for predicting the efficacy of novel therapies. Gurevich and coworkers developed a specific differentiation protocol for the generation of cryopreservable hepatocytes from a panel of induced pluripotent stem cells from humans with NASH [136]. They found that the hepatocytes from donors with NASH successfully maintained and reproduced steatosis. They also reported that these hepatocytes were able to be integrated into 3D liver organoids, together with iPSC from NASH patients differentiated into analogs of Kupffer cells and hepatic stellate cell precursors, with the maintenance of hepatic function for at least 10 days [136]. Thus, this study highlighted a powerful new model based on cocultures of cells derived from NASH patients that mimics some of the hallmarks of the disease, which could help to guide general and personalized treatments. Using a similar approach, Raabe’s group demonstrated that irreversibly damaged livers from NASH patients consistently give rise to long-term expandable bipotent ductal organoids that readily undergo hepatic differentiation and functionally reproduce inflammation and fibrosis [137]. The combination of 3D structures of this type with methylomic, metabolomic, and transcriptomic analyses may make it possible to implement organoid-based phenotyping in the personalization of disease modeling and drug development. Furthermore, efforts have been made to make use of organoid technology to develop new strategies for improving patient stratification, notably by considering the interaction between genetic and environmental risk factors. Hendriks et al. developed a scalable, personalized hiPSC-organoid platform for investigating the etiology of steatosis (e.g., exogenous (overload nutritional diet) and genetic origins (PNPLA3 I148M high-risk variant and monogenic predisposition to lipid disorders)), for use with a drug-CRISPR toolkit for NAFLD target identification and testing [132,138,139]. Kimura et al. proposed the development of an organoid-level “forward cellomics” platform [140]. They created a genetically diverse population organoid panel (POP) by mixing cryopreserved foregut progenitors from multiple donors. The POP was treated with FFA (oleic acid) to induce a steatohepatitis phenotype and the genotype–phenotype associations were analyzed “en masse” to capture the pathological genetic variation associated with NAFLD [140]. These approaches are innovative and very promising, but there is still considerable room for improvement. For example, there is a need for cocultures with nonparenchymal cells to extend the application of POP models and CRISPR, and drug-screening approaches for the later stages of the NAFLD-NASH spectrum.

Liver organoids are among the most advanced human-cell-based 3D liver models and their potential for use in NAFLD studies is considerable, as they can be used not only for patient- or gene-mutation-specific lipid metabolism studies, but also for large-scale drug testing to predict the clinical outcomes in personalized medicine and drug efficacy.

### 3.3. Liver-on-a-Chip

Until recently, spheroids/organoids were considered to be the 3D in vitro models of choice for studying NAFLD [60]. However, the automated control of critical parameters, such as temperature, fluid pressures, cell shear stress, nutrient supply, and waste removal, is not possible with these static platforms. Efforts have been made to overcome these limitations by producing powerful “organ-on-a chip” (OOC) platforms for dealing with these parameters and for the dynamic modeling of diseases and drug testing [141,142,143]. OOC systems bear some similarity to spheroids/organoids. They consist of hollow channels lined with living cells and tissues grown under a more tightly regulated environment, so as to reproduce organ-level and even whole body-level functions [141,144,145,146,147]. Through the integration of cell biology with microengineering and microfluidics, OOCs model physiological and pathological tissue microenvironments, thereby overcoming the limitations of conventional in vitro and in vivo approaches [147].

For the liver, such chips are generated by using hepatocytes to seed a polymeric scaffold consisting of tiny tubes, replicating the microarchitecture of the liver. The device is organized into a long-donut-shaped structure closely resembling a hepatic lobule, through which a microfluid can be passed to recreate the dynamic physicochemical environment of the liver, the gradients involved in zonation, and hepatic functions, rendering this device relevant as a model for studying NAFLD [101,148,149,150,151,152]. Gori and coworkers developed the first liver-on-a-chip (LOC) model by culturing HepG2 cells in a central chamber surrounded by closely spaced parallel microchannels, simulating endothelial cells to mimic the endothelial–parenchymal interface of a liver sinusoid [153]. More gradual and milder intracellular triglyceride accumulation is possible, with a higher hepatic cell viability than that in static 3D models, making it possible to more accurately reproduce the conditions of chronic steatosis observed in vivo. The exposure of this microfluidic device to FFA supplementation for 48 h has been shown to lead to a significant accumulation of intracellular lipids, indicating that this device can readily reproduce the establishment of steatosis [153]. The power of liver-on-chip analyses has been greatly increased since this initial work, through the integration of multiple cell types to reflect the complexity of the liver microenvironment and its role in NAFLD progression more accurately [148]. For example, Lasli and coworkers created a 3D device composed of HepG2 cells and HUVECs transferred onto a chip platform to establish a “steatosis disease-on-a-chip model” [154]. They demonstrated the reversibility of steatosis by treating their LOC device with antisteatotic drugs (metformin or pioglitazone); this treatment triggered a return of intracellular lipid concentrations to basal levels [154]. Du et al. developed a liver lobule chip based on human hepatocytes (HepaRG cells), LSECs, and HSCs that mimicked liver zonation; they demonstrated a change in lipid zonation within a single liver lobule during the early stages of NAFLD progression [149]. Moreover, the treatment of the device with obeticholic acid and elafibranor, which are known to have beneficial effects on lipid metabolism [155,156,157], was shown to prevent or reverse steatosis [149]. Freag and coworkers developed a NASH-on-a-chip model to investigate the NAFL/NASH transition; this model was based on coculturing the four major types of human primary liver cells (hepatocytes, Kupffer cells, liver sinusoidal endothelial cells, and hepatic stellate cells) under microfluidic dynamics and exposing the device to lipotoxic stimuli (FFAs with and without LPS) [151]. Not only did Freag and coworkers demonstrate that it was possible to maintain their microstructured liver tissue under disease-inducing conditions for at least 10 days, but they also showed that this led to the gradual development of the key phenotypic characteristics of human NASH, including the accumulation of intracellular lipids, hepatocellular ballooning, and the expression of inflammatory and profibrotic markers. Furthermore, the exposure of the chip to elafibranor inhibited the development of NASH-specific hallmarks [151].

As mentioned above, several genetic polymorphisms are associated with NAFLD risk [158]. As a means of investigating the effect of the PNPLA3 I148M mutation, Kostrzewski et al. cultured human hepatocytes, KCs, and mutated HSCs in perfused LOC platforms [159]. They observed that the IL-6 secretion in FFA-treated LOCs was enhanced by the presence of this mutation, thus demonstrating the utility of the LOC as a tool for predicting the effects of the genetic polymorphisms associated with NASH progression [159].

The interactions between multiple organs, such as those of the gut-adipose tissue-liver axis, play important roles in NAFL pathogenesis [160,161]. LOC complexity has increased in the last few years and researchers are now focusing their efforts on multi-organ platforms to connect different organs-on-a-chip, in order to assess the role of other tissues in NAFLD development. Lee and coworkers developed a gut-liver-on-a-chip device to reproduce the absorption of fatty acids in the gut and the subsequent accumulation of lipids in hepatocytes [162]. They showed that the presence of gut cells in their model modified drug efficacies with respect to the monoculture model, suggesting that the gut–liver chip can partially reflect the dynamic interactions of drugs with the gut and the liver, thereby improving the predictive value for drug efficacy [163]. Kamei’s group recently described an enhanced gut-liver-on-a-chip platform with integrated microvalves and a pump providing access to individual cell-culture chambers without undesirable cross-contamination, and closed circulation of the medium to mimic the human gut–liver axis [103]. Slaughter et al. developed an adipose tissue-liver-on-a-chip system consisting of both hepatocytes and white adipocytes, which they used to model NAFLD phenotypes in both the liver and adipose modules, together with crosstalk between the organs [164]. This model made it possible to explore the roles of adipocyte lipolysis and insulin resistance in NAFLD, and the exchange of cytokines and adipokines between organs [164].

Liver-on-a-chip systems are highly promising for studies of liver pathophysiology in the context of NAFLD. A large number of liver-on-a-chip models are currently commercially available [148] and, undoubtedly, future developments will endow these liver-on-a-chip systems with an even greater potential to improve our understanding of liver pathophysiology and for use in the development of treatments for NAFLD-related disease.

### 3.4. Precision Cut Liver Slices

Precision-cut liver slices (PCLS) have been used since the 1980s [165] as in vitro liver models with a tremendous potential to reproduce the complex multicellular histoarchitecture of the hepatic environment, including liver-infiltrating immune cells, whilst maintaining complex biochemical and molecular processes [166]. These slices are obtained by cutting fresh liver tissue with a Krumdieck tissue slicer or automated vibratome [167] to thicknesses of as little as 100 µm [168], although the most widely used thickness is 250 µm, generally with a diameter of 5–8 mm. These slices are cultured in regular tissue culture plates in static, dynamic, or bioreactor-based systems [104,167,169,170]. Liver slices are reproducible, cheap, and maintain the viability of hepatocytes, Kupffer, endothelial, and hepatic stellate cells for five days in controlled culture conditions [167,168], and up to 15 days under certain conditions [171]. Interestingly, one of the main advantages of PCLS cultures is that multiple readouts can be collected from a single slice, as both the slice itself and the culture medium can be analyzed [167]. The tissue for PCLS preparation is usually obtained via partial hepatectomy, from discarded surgical waste, explanted tissue, or non-transplantable tissue. PCLS cultures were initially used for metabolic studies and toxicity testing, but over the last 20 years, the focus of PCLS experiments has shifted towards studies on chronic liver disease, such as fibrosis [167,172]. Indeed, a spontaneous fibrogenic process has been reported to occur during the prolonged incubation of liver slices, as the procedure induces tissue repair and regenerative responses [172,173].

Some of the early processes leading to NAFLD can be investigated in slices from healthy human livers. Indeed, PCLS challenged by the addition of toxic fats (a mixture of oleic and linoleic acids) to the culture medium reproduce the hepatic steatosis, lipid deposition, and lipotoxicity observed in patients during the early stages of NAFLD progression [174]. Using hPCLS, Janssen and coworkers obtained proof-of-concept that PPARα, through its immunosuppressive/anti-inflammatory effect in the human liver, may be relevant to the treatment of non-alcoholic fatty liver disease [175]. Furthermore, the spontaneous induction of fibrogenesis observed in healthy PCLS facilitates evaluations of the efficacy of antifibrotic compounds [176]. Interestingly, the use of this system has underlined clear differences in the fibrotic process and the efficacy of antifibrotic compounds between species, explaining why several drugs with proven antifibrotic effects in animal studies are not effective in humans [176,177]. Aoudjehane and coworkers went further, generating viable functional steatotic human PCLS [178] with a view of improving the quality of so-called “marginal” liver grafts, such as steatotic liver grafts. They demonstrated the efficacy of a “degreasing” cocktail for decreasing the number of intracellular fat droplets, TG content, ER, and oxidative stress in these steatotic hPCLS, thus paving the way for efforts toward increasing the number of usable liver grafts [178].

Finally, the wider use of hPCLS, reflecting inter-individual hepatic heterogeneity, would improve the prediction of treatment efficacy and might also provide insight into the factors (e.g., genetic and epigenetic) contributing to this heterogeneity [179].

## 4. Conclusions and Perspectives

NAFLD-HCC is undoubtedly a growing global health problem and its contribution to HCC morbidity and mortality is likely to escalate in the coming decades. However, due to the intricate and multifaceted pathophysiology of NAFLD, finding an ideal animal model that can comprehensively mimic the complete spectrum of NAFLD within a reasonable timeframe is challenging. In recent years, many excellent (in vitro/in vivo) models of NAFL/NASH-HCC have significantly advanced our understanding of the mechanistic basis of NAFL/NASH-HCC and have identified novel biomarkers, prognostic markers, and candidate treatment targets. Although in vitro models still require further technological advancements and cost reductions, their continuous improvement holds promise for achieving a comprehensive understanding of the pathogenesis of human NAFL/NASH-HCC and expediting the translation of basic scientific findings into clinical breakthroughs. As shown in this comprehensive review of various preclinical models, specific advantages and disadvantages are inherent to each mouse model (Table 1), and these models reproduce different aspects of human disease. Furthermore, the combination of in vitro and in vivo models could serve as a viable approach to accumulating sufficient knowledge, greatly assisting in understanding liver diseases, the development of new therapies, and the advancement of personalized medicine in hepatology. Accordingly, investigators should use their understanding of the disease they wish to study to ensure that they select the most appropriate model for their research objectives.

## Figures and Tables

**Table 1:** Summary of the most relevant models of NAFL/NASH/HCC.

Model Type or Name	Phenotype					Fibrosis	NASH	Human NASH Gene Signature	Time to Disease	Subsequent Development of HCC	Advantage	Disadvantage	References
	Insulin Resistance	Obesity	Steatosis	Inflammation/ER Stress	Ballooning								
**NAFLD**													
**HFD**	YES	YES	Strong	Weak	NO	Slight	NO	NO	Translocation of bacteria in 1 week, fibrosis after 9 weeks, hepatic inflammation after 19 weeks	No; requires additional insult	Low costs; easy to operate	Requires large sample size; difficult comparison between groups and protocols related to various dietary compositions	*Ito et al., 2007; Nakagawa et al., 2014; Flessa et al., 2022* [44,49,50]
***ob/ob* (point mutation in leptin gene)**	YES	YES	Strong	Weak	NO	Second insult	NO	ND	First signs of obesity recognizable at 4 weeks of age	No; requires additional insult	Features similar to human NAFLD	Leptin deficiency does not seem to play a role in NAFLD and NASH development in humans; Do not develop NASH or fibrosis without second insult	*Kristiansen et al., 2016* [55]
***db/db* (point mutation in leptin receptor)**	YES	YES	Strong	Weak	NO	Second insult	NO	ND	Develop NASH after the addition of MCD diet for 2 weeks	No; requires additional insult	Features similar to human NAFLD	Leptin deficiency does not seem to play a role in NAFLD and NASH development in humans; Do not develop NASH or fibrosis without second insult	*Trak-Smayra et al., 2011* [61]
**NASH**													
**MCD**	NO	Weight loss	Strong	Strong	YES	YES	YES	ND	Steatohepatitis after 10 days and fibrosis after 8–10 weeks	No; requires additional insult	Short period; Easy to operate; High reproducibility, appropriate for the study of fibrosis mechanisms	No NAFLD-related metabolic syndrome; severe weight loss and liver atrophy	*Caballero et al., 2010; Itagaki et al., 2013* [65,66]
**CDAA**	NO	Weight loss	Strong	Medium	YES	YES	YES	ND	Mild to moderate fibrosis ~22 weeks	~25% prevalence of HCC 84 weeks	Robust; reproducible	Long-period; high costs; age-related systemic changes, No advanced fibrosis; Disease etiology does not mimic humans	*Kodama et al., 2009; Denda et al., 2007; Matsumoto et al., 2013* [76,77,78]
**High-Fructose Diet**	YES	YES	Strong	Medium	YES	YES	YES	YES	12~16 weeks can have fibrosis and NASH	Need other diet	Recapitulates histopathological characteristics of human from NAFLD to NASH	Do not develop HCC without second insult	*Kohli et al., 2010; Nigro et al., 2017* [80,81]
**ALIOS**	YES	YES	Strong	Medium	YES	YES	YES	ND	NASH 12–16 weeks versus late NASH 24–30 weeks	Not relevant	Recapitulates histopathological characteristics of human NASH	No longer available as trans fats cannot be included in food	*Tetri et al., 2008; Trevaskis et al., 2012; Gallage et al., 2022* [43,84,85]
**NASH-HCC**													
**CDA-HFD**	NO	NO	Strong	Weak	YES	YES	YES	ND	Lipid droplets with infiltration of inflammatory cells after 1 week;enlarged fatty liver with fibrosis in 6 week; NASH ~12 weeks	24–36 weeks: 100% of mice	Accelerated model of NASH with severe fibrosis. High induction of hepatocellular adenomas and carcinomas	Does not induce obesity or metabolic syndrome	*Matsumoto et al., 2013; De Minicis et al., 2014; Ikawa-Yoshida et al., 2017* [76,87,88]
**CDHFD**	YES	YES	Strong	Weak	YES	YES	YES	ND	NASH 16~18 weeks; HCA~20 weeks	12 months: ~25–30% of mice; 15 months: ~50–70% of mice	ROS production, lipid peroxidation, and mitochondrial dysfunction. Fibrosis around central vein. Overexpression of cytokines TNF-α and IL-6	Lack of choline is less physiological. Lower degree of fibrosis compared to WDs	*Wolf et al., 2014; Pfister et al., 2021; Malehmir et al., 2019* [35,39,92]
**WD+CCl4**	NO	NO	Strong	Strong	YES	YES	YES	YES	Microbiome remodeling within 12 weeks; F4 fibrosis and HCC ~24 weeks,	24 weeks: 100% of mice	Recapitulates histopathological and transcriptional characteristics of human NASH	Does not induce obesity or metabolic syndrome.	*Tsuchida et al., 2018* [93]
**Mup-upA +HFD**	YES	YES	Strong	Medium	YES	YES	YES	YES	Ballooning hepatocytes, pericellular and bridging fibrosis ~24 weeks; small HCC ~32 weeks	~32–40 weeks: ~80% of mice	Can spontaneously mimic Human NASH to HCC; does not rely on the administration of liver toxins or carcinogens	Genetic background	*Nakagawa et al., 2014; Febbraio et al., 2019* [40,50]
**DIAMOND**	YES	YES	Strong	Weak	YES	YES	YES	YES	Mice develop steatosis within 16 weeks; NASH ~16–24 weeks and nodule formation by 52 weeks (90%)	52 weeks: 90% of mice	Histology and transcriptome mirror human NASH	Genetic background of DIAMOND mice is unique, making it difficult to cross them with other gene targeted mice	*Asgharpour et al., 2016* [98]
